# Letter to editor: The waist circumference-adjusted associations between hyperuricemia and other lifestyle-related diseases: methodological issues in cross-sectional study

**DOI:** 10.1186/s13098-017-0223-3

**Published:** 2017-04-11

**Authors:** Shiva Mansouri Hanis, Fatemeh Khosravi Shadmani, Kamyar Mansori

**Affiliations:** 1 Social Determinants of Health Research Center, Kurdistan University of Medical Sciences, Sanandaj, Iran; 2 Modeling in Health Research Center, Institute for Futures Studies in Health, Kerman University of Medical Sciences, Kerman, Iran; 3 Social Development & Health Promotion Research Center, Gonabad University of Medical Sciences, Gonabad, Iran; 4 Department of Epidemiology, School of Public Health, Iran University of Medical Sciences, Tehran, Iran

**Keywords:** Methodological issues, Cross-sectional study, Prediction

## Dear Editor-in-Chief

We studied the article written by Miyagami et al. [1] that published in Diabetology & Metabolic Syndrome journal in February 2017. The aim of this study was the relationship between hyperuricemia and lifestyle-related diseases after adjusting with waist circumference (WC). Finally, the authors of this study concluded that Hyperuricemia is an independent predictor of several lifestyle-related diseases, even after adjusting for age, WC, and lifestyle in both sexes which is closely related with insulin resistance. Hyperuricemia might require greater attention during the prevention of lifestyle-related diseases and future cardiovascular disease [1]. However, although this was an appropriate research and its results were very interesting, some methodological issues should be considered.

1. Miyagami et al. [1] evaluated the predictive performance hyperuricemia on several lifestyle-related diseases in a cross-sectional study, whereas longitudinal researches are necessary for making assumptions in clinical prediction models [2]. In other words, assurance of the temporality assumption presence (the dependent variable has to occur after the independent variable) is essential in the prediction model. Thus, prediction models resulting from cross-sectional designs can be misleading [2, 3].
2. Considering the predictive performance of hyperuricemia on several lifestyle-related diseases is an optimistic interpretation. The internal and external validation of the prediction model must be done through bootstrapping and split-validation, respectively [4].

Therefore, according to the above explanation, it is necessary considering to this point in interpretation of results of this study for readers.

## References

[1] Miyagami T, Yokokawa H, Fujibayashi K, Gunji T, Sasabe N, Okumura M (2017). The waist circumference-adjusted associations between hyperuricemia and other lifestyle-related diseases. Diabetol Metab Syndr.

[2] Steyerberg E (2008). Clinical prediction models: a practical approach to development, validation, and updating.

[3] Pakzad R, Safiri S (2017). Letter to the editor: phosphorus as predictive factor for erectile dysfunction in middle aged men: a cross sectional study in Korea; methodological issues to avoid prediction fallacy. Investig Clin Urol.

[4] Noto D, Cefalù A, Barbagallo C, Ganci A, Cavera G, Fayer F (2016). Baseline metabolic disturbances and the twenty-five years risk of incident cancer in a Mediterranean population. Nutr Metab Cardiovasc Dis.

